# Perspectives and relevance of evidence-based medicine in Brazil*

**DOI:** 10.1590/1516-3180.2018.1365231018

**Published:** 2018-10-22

**Authors:** Álvaro Nagib Atallah, José Luiz Gomes do Amaral

**Affiliations:** I Titular Professor and Head of the Discipline of Emergency Medicine and Evidence-Based Medicine, Universidade Federal de São Paulo (UNIFESP); Co-founder of the International Cochrane Collaboration and Director of Cochrane Brazil; Director-Elect of the International Cochrane Collaboration; and Editor-in-Chief of the São Paulo Medical Journal and the journal Diagnóstico & Tratamento (2017-2020). Director of the Centro Brasileiro de Saúde Baseada em Evidência (CBSBE).; II Titular Professor of the Discipline of Anesthesiology, Pain and Intensive Therapy of the Department of Surgery, Escola Paulista de Medicina - Universidade Federal de São Paulo (EPM-UNIFESP); and President of Associação Paulista de Medicina for the three-year period 2017-2020. Editor of the São Paulo Medical Journal and the Journal Diagnóstico & Tratamento (2017-2020).

Scarcity of resources and their inefficient use form the basis of the difficulties of the Brazilian healthcare system. Practical application of evidence-based medicine (EBM) is grounded in identifying the best scientific evidence regarding the efficacy, effectiveness, efficiency and safety of every intervention, whether it is of diagnostic, therapeutic or preventive nature. Efficiency refers to doing more technically with fewer resources; efficacy refers to doing this under ideal conditions; and effectiveness refers to doing this in contexts that take into account patients’ real conditions and their circumstances. In turn, safety requires proper proof and assurance that every decision will bring more benefit than harm to individuals and to society.

There is absolutely no doubt that the impact of EBM has improved efficiency within the field of healthcare worldwide. In Brazil too, adoption of the concepts of EBM by the Ministry of Health has been shown to avoid waste, of the order of tens of billions of reais, and has brought major benefits for the population.^1^^,^^2^ Moreover, this movement has contributed greatly towards the development of basic research and research applied to clinical medicine. This has been translated into better results from finite investments in scientific investigations in the field of healthcare. This must not remain unnoticed within Brazilian research funding agencies.

It is therefore of paramount importance to stimulate teaching of EBM within undergraduate courses within the field of healthcare. In Brazil, fewer than 20% of medical schools include EBM among the concepts forming part of the curriculum!^3^ It is rare for medical schools to have a sector for EBM, yet this is fundamental for its propagation and implementation. EBM has immense value for constructing critical awareness regarding healthcare interventions in the initial phases of the course, such that students will gain the ability to better analyze scientific communications, whether these are in published data or at congresses in which they participate. From the time when students start to do research, they will incorporate the fundamentals of EBM into a wide variety of fields of healthcare and will translate their knowledge into technical production.

Furthermore, over the last few years, hundreds of postgraduate students of EBM have earned their degrees in Brazil. These professionals comprise not only doctors but also psychologists, nurses, dentists, physiotherapists, librarians, journalists, lawyers, prosecutors, biologists, pharmacists, speech therapists and many others. In our setting, we now have a critical mass with the capacity to start to disseminate this culture and equip our teaching institutions. We need to move forward in this regard, given the potential benefits of EBM for improving our social structure.

Technology and innovation are the buzzwords today. However, there is a need for careful analysis on the proof (or evidence), to demonstrate that incorporation of these technologies and innovations is efficient and safe. Rationality in acquiring new technologies is a fundamental premise, particularly when application of public resources is involved.

For more than two decades, the São Paulo Medical Association (Associação Paulista de Medicina) has focused its attention on EBM. It has striven towards consolidating the culture that this movement represents, and has done so not only for the benefit of Brazilian medicine but also, above all, to ensure full rights to quality healthcare for our population, in other words, evidence-based healthcare rights and implementation.
